# Long-Term Outcomes of Sacrococcygeal Germ Cell Tumors in Infancy and Childhood

**DOI:** 10.1155/2015/398549

**Published:** 2015-10-04

**Authors:** Rangsan Niramis, Maitree Anuntkosol, Veera Buranakitjaroen, Achariya Tongsin, Varaporn Mahatharadol, Wannisa Poocharoen, Suranetr La-orwong, Kulsiri Tiansri

**Affiliations:** ^1^Department of Surgery, Queen Sirikit National Institute of Child Health, Bangkok 10400, Thailand; ^2^College of Medicine, Rangsit University, Bangkok 10400, Thailand

## Abstract

*Purpose*. The aim of this study was to evaluate long-term outcomes of sacrococcygeal germ cell tumors (SC-GCTs) over a 15-year period.* Materials and Methods*. A retrospective review was conducted of all pediatric patients treated for SC-GCTs at our hospital from 1998 to 2012.* Results*. Fifty-seven patients were treated for SC-GCTs with the most common in Altman's classification type I. Age at surgery ranged from one day to 5.6 years. Tumor resection and coccygectomy were primarily performed in about 84% of the cases. Pathology revealed mature, immature, malignant sacrococcygeal teratomas (SCTs), and endodermal sinus tumors (ESTs) in 41 (72%), 4 (77%), 6 (10.5%), and 6 (10.5%), respectively. Recurrence of discase occurred in 3 of 41 patients with mature teratomas (7.3%); 2 recurrences with mature teratomas and one recurrence with EST. Five of 6 malignant SCTs and 3 of 6 ESTs responded well to the treatment. Alpha-fetoprotein (AFP) level was elevated in both malignant teratomas and ESTs. No immediate patient death was noted in any of the 57 cases, but 4 patients with malignant tumors and distant metastasis succumbed at home within 2 years of the initial treatment.* Conclusion*. Benign SCTs have a significant recurrence rate of approximately 7%. Close follow-up with serial AFP level monitoring should be done for 5 years after initial tumor resection and coccygectomy. The survival rate for malignant SC-GCTs with distant metastasis was unfavorable in the present study.

## 1. Introduction

**Figure 1:**
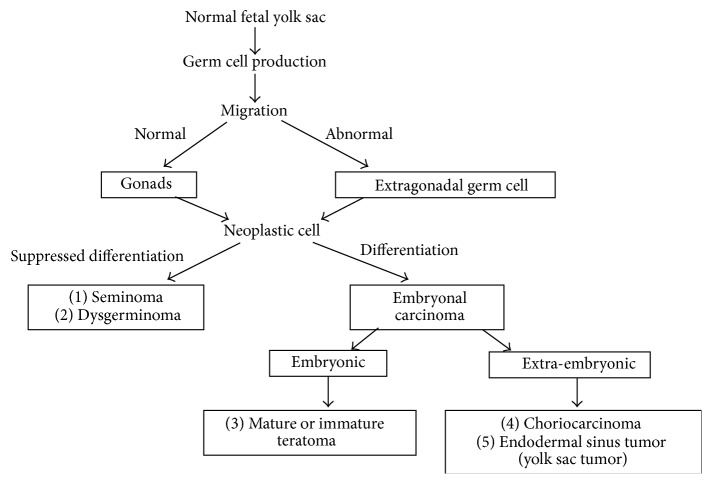
Development of germ cell tumors.

Pediatric germ cell tumors (GCTs) are neoplasms derived from primodial germ cells and may occur both inside the gonads and in extragonadal organs. The five main histologic categories of GCTs are dysgerminomas (in the ovary), seminomas (in the testes), teratomas, choriocarcinomas, and endodermal sinus tumors (ESTs) or yolk sac tumors [1, 2] (Figure 1). The most common site of extragonadal GCTs in the pediatric population is the sacrococcygeal region followed by the anterior mediastinum, intracranial region, retroperitonium, neck, stomach, and vagina [2]. The most common sacrococcygeal germ cell tumors (SC-GCT) are teratomas which mostly behave as benign tumors and, less commonly, as malignant ones [1–3]. The least common SC-GCT is EST which characteristically presents as a malignant tumor. Sacrococcygeal teratomas (SCTs) are classified as mature, immature, and malignant forms [4–6]. A mature SCT is a benign tumor containing only mature teratomatous components while immature SCTs contain immature tissues which are not frankly malignant, and malignant SCTs are composed of mature or immature teratomatous tissues and any of the other 4 malignant GCTs. The behavior of SCT does not depend entirely on histology appearance, but also on the age of patient at surgery. This paper presents a study of a single tertiary institute for pediatrics in order to evaluate long-term outcomes of SC-GCT over a 15-year period.

## 2. Materials and Methods

After the study protocol had been approved by the Institutional Reviewer Board (Document number 57-054), medical records were reviewed of all the patients with SC-GCT treated at Queen Sirikit National Institute of Child Health between January 1998 and December 2012. Data were obtained on gestational age (GA), birth weight (BW), age at operation, anatomical types, pathological reports, and results of treatment. The tumors in this region were classified into 4 anatomical types as described by Altman et al. [7]. SCT was categorized into 3 histologic types: mature, immature, and malignant teratomas [4–6]. If a pathologic report revealed one type of malignant GCT without mentioning the presence of teratomatous components, the tumor was diagnosed as a malignant GCT in accordance with the histologic description. The tumor was diagnosed as mixed GCT if it contained more than one type of malignant GCT. The patients were investigated preoperatively by plain film of the pelvis, ultrasonography, computerized tomography (CT) scan, or barium enema in some cases. Preoperative and postoperative alpha-fetoprotein (AFP) levels were determined in all of the patients. An AFP level of 20 ng/mL or lower was considered to be normal in children over 8 months of age [8, 9]. The Pediatric Oncology Group (POG) and Children Cancer Group (CCG) staging of malignant extragonadal GCT was used for analysis of the malignant cases (Table 1). Adjuvant therapy for malignancy was recorded and long-term follow-up of clinical outcomes was evaluated.

## 3. Results 

A total of 57 patients were treated for SC-GCTs during the study period. There were 13 males and 44 females; hence the male to female ratio was approximately 1 : 4. Six patients with SC-GTCs were born at Rajavithi Hospital (previously known as the “Women's Hospital”) during the period in which there were 131,851 live births. Therefore, the incidence of SC-GCT at Rajavithi Hospital was 1 : 21,975 live births. The tumors were identified prenatally by ultrasonography, at birth and later in infancy and childhood in 13 (22.8%), 26 (45.6%), and 18 (31.6%) cases, respectively. Average BW was 3250.9 ± 410.5 grams (range from 2700 to 4610 grams) and average GA was 39.1 ± 1.7 weeks (range from 35 to 40 weeks). Three cases had tumor ruptures since birth; 2 of these were cases of vaginal delivery and one of cesarean section. Of the 15 patients diagnosed at over one year of age, the presenting symptoms included constipation, dysuria, palpable suprapubic mass, and abdominal pain in 12, 9, 8, and 3 cases, respectively. Two cases with coccygeal pain and one case with weakness of the lower extremities were identified as having lung and vertebral metastasis at diagnosis.

**Figure 2:**
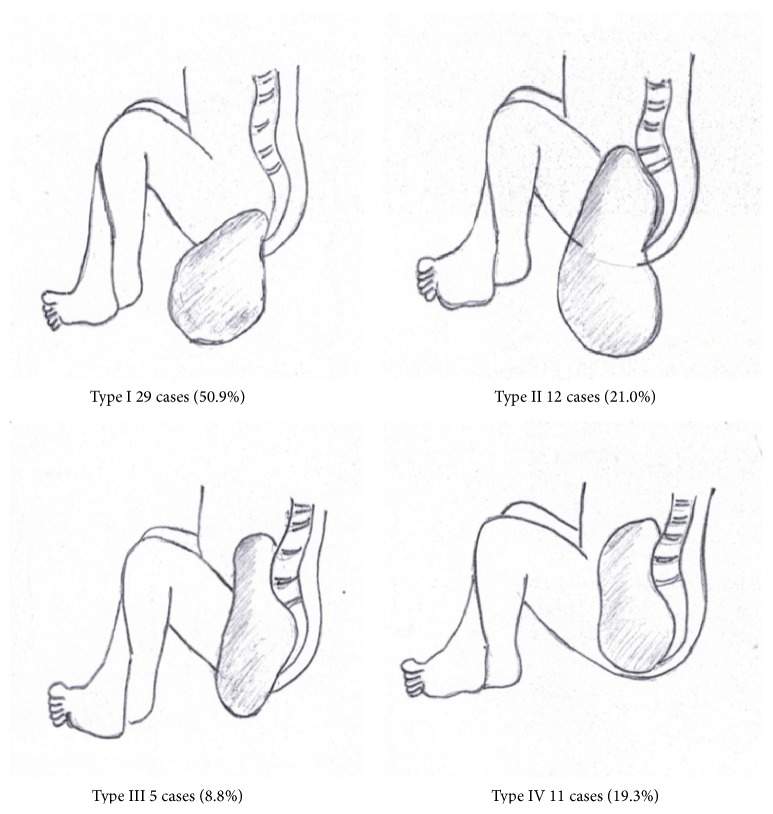
Classification system for 57 sacrococcygeal germ cell tumors based on Altman's American Academy of Pediatrics series [7].

Age at surgery ranged from one day to 5.6 years (median 43 days, mean 281.9 ± 439.6 days). For the initial treatment, total tumor resection with coccygectomy was performed in 47 cases, while tumor biopsy was done in 8 cases and partial excision in the other 2. The total 57 cases of SC-GCT were customarily grouped into 4 anatomical types according to Altman's classification [7]. Twenty-nine patients (50.9%) were categorized as type I, 12 (21.0%) as type II, 5 (8.8%) as type III, and 11 (19.3%) as type IV (Figure 2). Pathological examination revealed 41 (72.0%) mature teratomas, 4 (7.0%) immature teratomas, 6 (10.5%) malignant teratomas, and 6 (10.5%) pure ESTs. The risk of malignancy varied from zero in type I to approximately 73% in type IV (Table 2). The relation between risk of malignancy and age at surgery is shown in Table 3. Patients who underwent surgery at or before one year of age had a risk of malignancy of only 2.41% (one in 42 cases) and the risk increased to 73.3% (11 in 15 cases) if the patients underwent surgery at the age of over one year. The youngest malignant case was 1.5 months of age at surgical excision with pathological report of malignant SCTs (including mature teratomas and ESTs).

**Figure 3:**
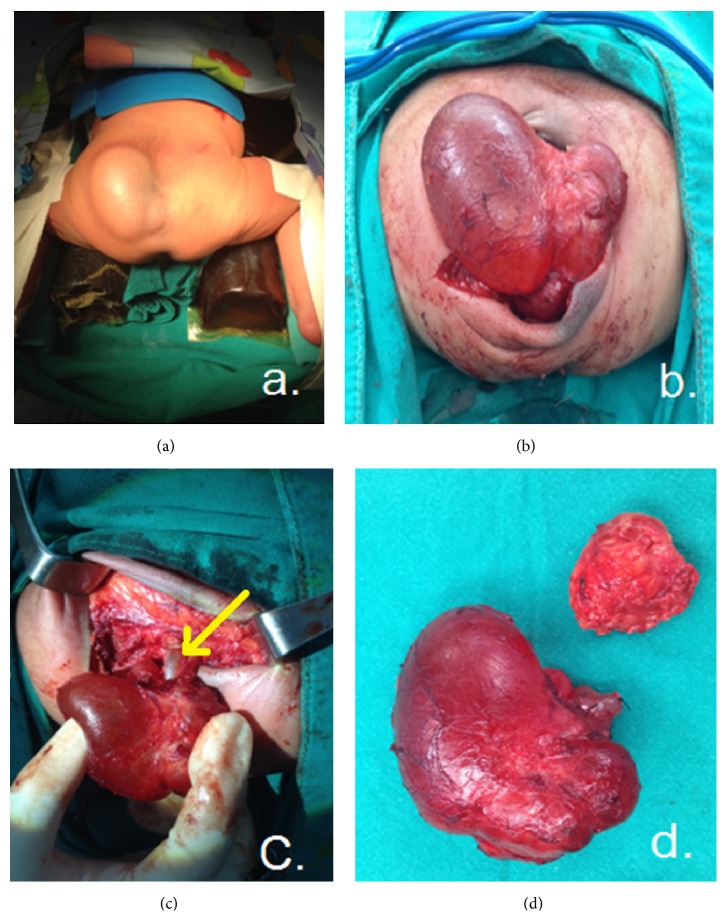
Resection of a type I sacrococcygeal teratoma; (a) the patient in frog-legged position (b) mobilization of the tumor, (c) exposure of the coccyx, and (d) removal of the tumor and coccyx.

Of the 41 cases (9 males and 32 females) with mature SCTs, age at surgery ranged from one day to 5.6 years. Thirty-three patients who underwent surgical resection at under 8 months of age had AFP levels ranging from 125.6 to 179,185 ng/mL/. The AFP level declined to normal limits (<20 ng/mL.) at the age of over 8 months after operation. Eight cases aged over 8 months at surgery had normal AFP levels (range from 0.6 to 4.8 ng/mL). All of the 41 patients with mature teratomas underwent tumor resection and coccygectomy (Figure 3). Tumor spillage during surgical excision was noted in 6 cases (14.6%). Three cases with tumor type IV required both a transsacral approach and abdominal laparotomy for removal of the intrapubic mass and coccygectomy. Recurrent disease developed in 3 of the 41 patients (7.3%) with mature teratomas between 3 months and 3.5 years after the initial resection. All of the recurrences were tumors initially categorized into Altman's type I with complete tumor resection and coccygectomy, but there were the evidences of tumor spillage during operation. Two of the recurrences were mature teratomas that were noted on rectal examination at 3 and 8 months after operation and treated with surgery alone. AFP levels at the recurrence period were within normal limits (1.5 and 2.5 ng/mL), and both of these patients are long-term survivors. One of the recurrences was found to be EST in a patient who presented with a presacral mass, a rising AFP level of over 2,000 ng/mL, and lung metastasis proven by a CT scan at 3.5 years after initial operation. The treatment included transsacral tumor biopsy, adjuvant chemotherapy (cisplatin, etoposide and bleomycin-PEB), and pelvic radiation and after 3 months; AFP levels declined to 2.6 ng/mL. At the last 6-year follow-up (9.6 years of age), the patient was doing well, with normal AFP levels and no evidence of lung metastasis from pulmonary CT scan (Table 4).

The tumors of 4 patients (one male and 3 females) with immature teratomas were categorized into type I in 3 cases and type II in the other case. Age at operation ranged from 3 to 45 days with AFP levels ranging from 1,000 to 50,000 ng/mL and declining to normal levels at the age of one year. Histology revealed premature teratomas of both grades I and II in 2 cases. No evidence of recurrence was noted at the follow-up of an average of 18 months.

Of the 6 cases (2 males and 4 females) with malignant SCTs, age at operation ranged from one month to 2.25 years with Altman's types II, III, and IV in 2, 1, and 3 case, respectively. AFP levels at operation ranged from 20,000 to 60,500 ng/mL. The initial surgical procedures included tumor biopsy in 3 cases and tumor resection with coccygectomy in the other 3 cases. Pathological examinations revealed immature teratomas with ESTs in 2 cases and mature teratomas with ESTs in the other 4 (Table 4). After surgery, 4 patients received adjuvant chemotherapy (PEB) and radiotherapy. Two patients, one in stage I and one in stage IV, received only chemotherapy. The patient with lung metastasis at diagnosis did not respond to chemotherapy treatment; his serum AFP level was still high at over 20,000 ng/mL, and he was lost to follow-up approximately one year after surgery and died at home. Five patients followed up for between 3 and 10 years after surgery (average 7 years); and they had a good response to adjuvant therapy, with AFP levels declining to normal levels within 3 months of chemotherapy and radiation. Three of the 5 patients underwent second-look operation for resection of the residual tumors; pathology revealed only mature teratomas in 2 cases and necrotic tissue without tumor cell in the other case.

Of the 6 patients (one male and 5 females) with pure ESTs, age at surgery ranged from 1.1 to 3.2 years with type III (2 cases) and type IV (4 cases). Two patients had evidences of lung metastasis at diagnosis and AFP levels before surgery ranged from 20,000 to 51,260 ng/mL. Their chief complaints included dysuria, constipation, palpable suprapubic mass, abdominal pain, coccygeal pain, and weakness of the lower extremities. Tumor biopsy alone was performed in 5 cases and partial tumor resection with coccygectomy was carried out in the other case. Adjuvant chemotherapy and radiotherapy were added after surgery. One patient developed colonic and bladder neck obstructions within one month of tumor biopsy and she required abdominal laparotomy, right transverse colostomy, and suprapubic cystostomy. Four patients underwent second-look operation with tumor resection and coccygectomy between 4 and 6 months after chemotherapy and radiation; pathology of the second operation showed benign fatty tissue, cartilage, and cell debris in all of 4 cases. Three of the 6 patients with pure ESTs did not respond to treatment and had tumor relapse with lung and brain metastases. They were lost to follow-up within 2 years of the initial treatment and succumbed at home. The remaining 3 cases responded to treatment, and they were doing well at the 3-year follow-up with normal AFP levels and no evidence of relapse or metastasis (Table 4).

## 4. Discussion 

The incidence of sacrococcygeal tumors at Rajavithi Hospital, Bangkok, Thailand, was approximately 1 : 22,000 live births, whereas the incidence of this entity had earlier been reported as 1 : 28,500 to 35,000 live births [10, 11]. As in previous reports, the present study revealed a female predominance with a 1 : 3 to 1 : 4 ratio [2–7, 11, 12]. SC-GCTs usually occur in patients in two clinical patterns: 1, neonates presenting with large benign tumors of mature or immature teratomas, and 2, infants and children presenting with primary malignant SCTs or pure ESTs located in the pelvis, including benign SCTs in some cases. SC-GCTs in neonates frequently present with the characteristic mass protruding from the sacrococcygeal region which can be detected by prenatal ultrasonography. Infants with sacral masses that are prenatally diagnosed as greater than 5 cm in size should be considered for abdominal delivery to avoid dystocia and tumor rupture [13]. Three of our patients were noted to have tumor rupture, even though one case was delivered by cesarean section. SC-GCTs in older infants and children generally have no protruding mass noted at birth. They usually have the clinical presentations related to bladder or rectal compression and a palpable suprapubic mass, and these features were found in 12 of the 15 patients of this study. These tumors presumably arise from normal germ cells deposited in the sacrococcygeal area that undergo malignant transformation or from unrecognized small foci of malignancy present at birth which eventually become the major tissue type in the tumor [14, 15].

The risk of malignancy of sacrococcygeal tumors is related to its anatomical type and age of patient at surgery. Type I tumors have the lowest risk and type IV have the highest risk of malignancy. This was well demonstrated in our series, in keeping with the report of Altman et al. [7]. In the American Academy of Pediatrics survey, the incidence of malignancy was 7–10% in patients operated upon at the age of less than 2 months but 48–67% if they were treated after 2 months of age [7]. In our present study, the incidence of malignancy was only 2.4% in patients who underwent surgery at the age of less than one year and 73.3% in the patients operated upon after one year of age. These findings indicated that SC-GCTs should be surgically treated as soon as possible after birth.

In some cases with a large size tumor of types III and IV, a complete resection requires a combination of abdominal and sacral approach, and the coccyx is removed along with the tumor in every case. Failure to remove the coccyx results in a high recurrence rate [15–17], and Gross et al. [16] reported a recurrence rate as high as 37% when the coccyx was not removed. The other factor associated with recurrence of SC-GCTs is spillage of tumor during surgery [18]. The present study noted a 7.3% recurrence rate with mature SCTs where the coccyx had been completely removed with tumor spillage during surgical resection in all of the patients with tumor recurrence. Many studies have demonstrated a recurrence rate of 10–21% after resection of neonatal sacrococcygeal tumors and incidences of recurrence occurring within 3 years [12, 19]. The longest period to recurrence was reported by Mahour et al. [4] with local recurrence of mature SCT at 4.5 years after initial resection of the previous mature SCT. The recurrent tumor is usually composed of mature teratomatous tissue; however, instances of malignant recurrence after previous resection of a mature SCT have been reported [4–6, 12, 17]. Recurrence with EST in one of our 3 patients occurred as late as 3.5 years after operation of mature SCT; this may possibly be due to the presence of malignant components in the primary tumor which were not recognized during histologic examination as a result of sampling error in a large tumor. Incomplete resection of malignant components may result in tumor recurrence.

Results of treatment of mature and immature SCTs were satisfactory in this study. There was no immediate postoperative death and no long-term sequelae of anorectal function (constipation and soiling) or urinary function (neurogenic bladder and incontinence) as in some reports [10, 20]. In contrast, treatments of malignant SC-GCT obtained unsatisfactory outcomes, especially sacrococcygeal EST or yolk sac tumor. Our patients with malignant SC-GCTs did not respond to adjuvant chemotherapy and radiotherapy; they developed distant metastases to the lung, vertebra, and brain in 53.8% of cases and died at home in 30.8% of cases (Table 4), similar to those in other reports [4, 6, 12, 21]. Several studies have demonstrated markedly improved survival in malignant sacrococcygeal tumors with the use of new regimens of adjuvant chemotherapy such as PVB (cisplatin, vinblastine, and bleomycin), JEB (carboplatin, etoposide, and bleomycin), and PEI (cisplatin, etoposide, and ifosfamide) [18, 22, 23].

Close follow-up after resection of benign SCT should be done, including physical examination of the lower abdomen and buttock region, rectal examination, and serial AFP determinations. AFP levels are proven to be elevated in early infancy before gradually decreasing to normal levels at 8 months of age [8]. The data of the present study and others [16, 24–26] indicate that the presence of EST is associated with elevated serum AFP levels. Serial AFP examinations are useful for diagnosis, determination of the completeness of malignant tumor removal, tumor recurrence during the follow-up period, and formation of the prognosis [8, 22–27]. Based on our present study and other reports, monitoring of serum AFP level should be carried out every 3–6 months for more than 3 years because malignant recurrence clinically presents within 3.5 years of initial resection of mature SCT.

## 5. Conclusion

Teratomas are the most common GCTs of the sacrococcygeal region. Benign SCTs have a recurrence rate of approximately 7% after initial tumor resection and coccygectomy. Some cases of recurrence may present with malignant tumors and distant metastasis, and close follow-up with serial AFP monitoring should be done for 5 years. Even though chemotherapeutic regimens are more effective in this era, survival for malignant SC-GCTs with distant metastasis was not satisfactory in the present study.

## Figures and Tables

**Table 1:** Pediatric Oncology Group/Children Cancer Group (POG/CCG) staging for malignant extragonadal germ cell tumors.

Stage	Characteristics
I:	Complete resection at any site; coccygectomy for sacrococcygeal site; negative tumor margins; tumor markers positive or negative

II:	Microscopic residual; lymph nodes negative; tumor markers positive or negative

III:	Gross residual or tumor biopsy only; retroperitoneal nodes negative or positive; tumor markers positive or negative

IV:	Distant metastasis, including liver

**Table 2:** Anatomical types of sacrococcygeal germ cell tumors and risk of malignancy.

Altman's type	Sacrococcygeal teratoma			Endodermal sinus tumor	Total
	Mature	Immature	Malignant		
I	25	4	0	0	29 (50.9%)
II	10	0	2	0	12 (21.0%)
III	3	0	0	2	5 (8.8%)
IV	3	0	4	4	11 (19.3%)

Total	41 (72.0%)	4 (7.0%)	6 (10.5%)	6 (10.5%)	57 (100%)

**Table 3:** Age distribution and histologic types of sacrococcygeal germ cell tumors.

Age at operation (years)	Sacrococcygeal teratoma			Endodermal sinus tumor	Total
	Mature	Immature	Malignant		
0-1	37	4	1	0	42 (73.7%)
1-2	1	0	3	4	8 (14.0%)
2-3	1	0	1	1	3 (5.3%)
>3	2	0	1	1	4 (7.0%)

Total	41 (72.0%)	4 (7.0%)	6 (10.5%)	6 (10.5%)	57 (100%)

**Table 4:** Malignant sacrococcygeal germ cell tumors and results of the treatment.

Patient/gender/age at operation	Altman's type	Initial operation	AFP level (ng/mL) Histology	Initial staging	Adjuvant therapy	Second operation	Outcomes
Mature teratoma (MT) and recurrence with endodermal sinus tumor (EST)							

1 F 3.5 y (at the recurrence)	IV	Tumor biopsy	2,000 EST	IV (lung metastasis)	Chemotherapy + radiotherapy	None	6-year FU Survival-NED^*∗*^

Malignant teratoma							

1 F 2.1 y	II	Total resection + coccygectomy	20,000 MT + EST	I	Chemotherapy	None	3-year FU Survival-NED^*∗*^

2 F 1.2 y	IV	Tumor biopsy	2,000 MT + EST	III	Chemotherapy + radiotherapy	Total resection + coccygectomy	8-year FU Survival-NED^*∗*^

3 M 9 m	IV	Partial resection + coccygectomy	43,430 MT + EST	III	Chemotherapy + radiotherapy	Total resection	8-year FU Survival- NED^*∗*^

4 F 1.5 m	II	Partial resection + coccygectomy	6,985 MT + EST	III	Chemotherapy + radiotherapy	None	10-year FU Survival-NED^*∗*^

5 F 1.2 y	IV	Tumor biopsy	60,500 MT + EST	III	Chemotherapy + radiotherapy	Total resection + coccygectomy	7-year FU Survival-NED^*∗*^

6 M 3 y	III	Tumor biopsy	60,000 IMT^*∗∗*^ grade 3 + EST	IV (lung metastasis)	Chemotherapy + radiotherapy	None	Lung metastasis Death at home after one year treatment

Endodermal sinus tumor							

1 F 1.5 y	III	Partial resection + coccygectomy	20,000 EST	III	Chemotherapy + radiotherapy	None	Lung metastasis Death at home after 2-year treatment

2 F 1.1 y	III	Tumor biopsy	58,704 EST	III	Chemotherapy	Total resection + coccygectomy	Lung and brain metastases Death at home after 2-year treatment

3 F 2.3 y	IV	Tumor biopsy	34,144 EST	IV (lung metastasis)	Chemotherapy + radiotherapy	Total resection + coccygectomy	2-year FU Survival-NED^*∗*^

4 M 1.3 y	IV	Tumor biopsy	2,207 EST	III	Chemotherapy + radiotherapy	Total resection + coccygectomy	3-year FU Survival-NED^*∗*^

5 F 1.8 y	IV	Tumor biopsy	51,250 EST	IV (lung metastasis)	Chemotherapy + radiotherapy	Laparotomy, colostomy, and cystostomy	Lung, long bone,and vertebral metastases Death at home after 10-month treatment

6 F 3.2 y	IV	Tumor biopsy	30,763 EST	IV (vertebral metastasis)	Chemotherapy + radiotherapy	Laparotomy, partial resection, and coccygectomy	3-year FU Survival-NED^*∗*^

^*∗*^NED: no evidence of disease; ^*∗∗*^IMT: immature teratoma.
